# Self-Relevant Disgust and Self-Harm Urges in Patients with Borderline Personality Disorder and Depression: A Pilot Study with a Newly Designed Psychological Challenge

**DOI:** 10.1371/journal.pone.0099696

**Published:** 2014-06-23

**Authors:** Sawsan Abdul-Hamid, Chess Denman, Robert B. Dudas

**Affiliations:** 1 Department of Psychiatry, University of Cambridge, Cambridge, United Kingdom; 2 Complex Cases Service, Cambridgeshire and Peterborough Foundation NHS Trust, Cambridge, United Kingdom; 3 Behavioural and Clinical Neurosciences Institute, University of Cambridge, Cambridge, United Kingdom; Max Planck Institute of Psychiatry, Germany

## Abstract

**Background:**

Borderline personality disorder (BPD) is a common psychiatric condition associated with self-harm. Self-harm is poorly understood and there is currently no treatment for acute presentations with self-harm urges.

**Objectives:**

By using a new task (Self-relevant Task; SRT), to explore emotions related to one's own person (PERSON task) and body (BODY task), to study the correlations of these emotions, specifically disgust, with self-harm urge level changes, and to test the task's potential to be developed into an experimental model of self-harming for treatment trials.

**Methods:**

17 BPD patients, 27 major depressive disorder (MDD) patients, and 25 healthy volunteers performed the SRT. Emotion labels were extracted from task narratives and disgust and self-harm urge level changes measured by visual analogue scales. We used validated rating scales to measure symptom severity.

**Results:**

The SRT was effective at inducing negative emotions and self-harm urge changes. Self-harm urge changes correlated with borderline symptom severity. Post-task disgust levels on the visual analogue scales were higher in BPD patients than in healthy controls in the PERSON task, and higher than in both control groups in the BODY task. Changes in disgust levels during the task were significantly greater in the patient groups. Post-task disgust levels or changes in disgust were not associated with self-harm urge changes (except the latter in MDD in the PERSON task), but self-harm urge changes and disgust (but no other emotion) narrative labels were on a whole sample level.

**Conclusion:**

Although associations with the analogue scale measures were not significant, self-disgust reported in the narrative of patients may be associated with a higher probability of self-harm urges. Further research with larger sample sizes is needed to confirm this relationship and to examine whether reducing self-disgust could reduce self-harm urges. The SRT was effective and safe, and could be standardized for experimental studies.

## Introduction

Self-harm (SH) is poorly understood and there is currently no specific treatment for acute presentations with self-harm urges. The UK has one of the highest rates of SH in Europe at 400 per 100,000 population [1], having a major impact on the health and wealth of the nation. SH is one of the commonest reasons for accidents and emergency attendance in England and Wales, with an estimated 140–150,000 hospital presentations each year. Up to half of these are for repeat episodes [2]. Self-harm is the strongest predictor of suicide; those who self-harm are at 17 times higher risk of suicide. They also have 15 times higher risk of dying due to an undetermined cause or accidental poisoning, and an elevated risk of dying from a range of other health problems, relative to the population average [3].

Although the aetiology of SH is multifactorial, a significant proportion of those presenting with SH suffer from borderline personality disorder (BPD). BPD affects approximately 6% of the population [4] and is commonly associated with SH [5], [6]; [7], [8]. Self-harm is a broad term including both suicidal behaviour and non-suicidal self-injury (NSSI). Although important differences between these are increasingly recognized [9], in BPD patients both are particularly common and clinically often difficult to distinguish. Although suicidal and non-suicidal self-harm are only partially overlapping phenomena [10] there is evidence to suggest that body image, which is an area of investigation in our study, is a shared predictor of both behaviour [11], [12]. In the absence of evidence to support the use of any psychotropic medication [13], the current NICE guidelines [14] recommend treating the consequences of SH. Sadly, in 10% of BPD patients, SH leads to death [15]. Although long-term psychotherapy can help reduce SH behaviour in BPD in the long run [16], currently no specific treatment is available for acute presentations with SH, be it for urges or high risk of repetition following an act of SH, i.e. there is a pressing clinical need to develop one.

Studying the effect of acute interventions on self-harm urges (SHU) is not easy. SH is usually carried out in private [17] and most of the information we have is from retrospective accounts from patients who self-harm or from experiments using a script to remind the person of a previous act of SH of their own or a standardized script [18]. The resulting SHU level changes are hard to standardize. Therefore, finding a suitable research paradigm, such as an emotional challenge, would be helpful.

According to a review on SH [19], self-injury is most often performed to temporarily alleviate intense negative emotions or to express self-directed anger or disgust. In a study of motives for non-suicidal self-injury in BPD [20], apart from guilt, sadness, anger, anxiety, shame, and some other, more complex emotions, disgust was one of the negative emotions reported by patients. A study on trauma-related disorders [21] found increased levels of disgust for both BPD and PTSD, suggesting a link between childhood sexual abuse and disgust in BPD. This suggests a special role for disgust in SH behavior. SH may be a coping strategy to deal with trauma symptoms [22] and the association of traumatic experiences with disgust. Furthermore, we chose to place a special emphasis on disgust, because disgust seems to be different in BPD patients in some important aspects from the other emotions. Interestingly, unlike most other emotions, disgust did not seem to diminish after self-harming in the Kleindienst et al. study. The contention that disgust behaves differently in BPD is also supported by our own clinical experience; we found that BPD patients often seek out disgusting or gory stimuli from the environment, whilst they are not looking for sources of sadness or anxiety in the same way. We were particularly interested in disgust related to one's own body, because patients with BPD commonly describe themselves or their body as repulsive and as no source of pleasure and body regard has been shown to be an important risk factor for SH in previous studies [23].

A specific problem with investigations of emotional functioning in patients with BPD is the presence of depressive symptoms, which warrants, with any finding, checking for specificity to BPD. This is the reason why we included a clinical control group with major depressive disorder (MDD). It is important to note some other similarities between the two groups. Patients suffering with MDD also often describe negative feelings related to their person as a whole or their body. Depressed patients often suffer with poor self-esteem and some describe themselves as unappealing or even disgusting. Indeed, disgust, including that related to one's body and behaviour, has been demonstrated to mediate between dysfunctional cognitions and depressive symptomatology in depression [24], and other disgust-related anomalies have also be noted in depression [25]. Depressed patients may also have SH thoughts or urges. Indeed a study found that negative body regard has an indirect effect on SH behavior though depressive symptoms [23]. In summary, there appears to be a considerable overlap in symptomatology between BPD and MDD, which warrants checking for specificity. For this reason, we compared our BPD patients with both a healthy and a depressed group of participants.

This paper reports on our pilot findings with the Self-relevant Task (SRT), a task we have designed, as part of a larger project, in order to make BPD patients reflect upon the negative aspects of themselves and their body. We hypothesized that BPD patients would report a high level of negative emotions, including disgust, whilst doing so, and that these emotions would be associated with SH urges. By asking the participants to write a free narrative on their self-relevant thoughts and the emotions evoked by them, the task also provided a record of these emotions. A great advantage of extracting emotions from these narratives as opposed to using visual analogue scales is free-report that allows for an exploration of dominant emotions experienced by the participant free of bias introduced by suggestions of emotions to consider. We wanted to pay special attention to disgust related to one's own self, hence included a visual analogue scale to measure this.

Our main objectives in this study were to study the differences between the BPD and control groups in overall disgust levels and disgust reactivity and the association between these variables and self-harm urges. We were also interested in how narrative and VAS measures of disgust compared in sensitivity to the associations investigated. Further objectives were to explore the overall pattern of emotions generated during the narrative and, finally, to investigate the validity of the SRT as a safe experimental procedure for use in patient populations for inducing self-harm urge changes.

Based on the above, we specifically predicted that both overall disgust levels (**hypothesis 1**) and disgust reactivity (**hypothesis 2**) would be greater in the BPD group than in the control groups in both subtasks of the SRT. We expected to find correlations between overall self-relevant disgust (**hypothesis 3**) and disgust reactivity (**hypothesis 4**) and SH urges in our whole sample and specifically within the BPD group.

## Methods

### Participants

A total of 69 women participated in the study. 17 patients with borderline personality disorder (BPD), 27 with major unipolar depression (MDD) and 25 healthy volunteers (HV) were included according to strict criteria. We used structured diagnostic interview schedules, the MINI (Mini-International Neuropsychiatric Interview, [26]) and SCID-II [27] to establish the diagnoses required for inclusion and exclude volunteers who did not meet criteria. BPD patients were outpatients recruited via the Complex Cases Service (CCS), a specialized personality disorders unit, participants with unipolar depression by newspaper advertisements, and healthy participants from the MRC-CBU healthy volunteer panel and also via advertisements. All participants were interviewed by a psychiatrist (the last author of this paper) with expertise in personality disorders. BPD patients with current or past history of any formally diagnosed psychotic illness or current major depressive disorder, or dependence on a psychoactive substance, as per the MINI, were excluded. The presence of depressive symptoms (as opposed to a full-blown, co-morbid major depressive illness) did not lead to exclusion. The presence of other personality disorder traits, but not that of the full-blown disorder, was permitted. In the MDD group, any comorbid psychiatric conditions as per the MINI or SCID led to exclusion, but the presence of personality disorder traits, without the full-blown disorder, was permitted. In healthy volunteers, any history or presence of psychiatric or neurological illness led to exclusion. No participant had any history of epilepsy, serious head injury, serious medical conditions, physical problems requiring hospitalisation, or surgery in general anaesthesia in the previous 6 months. Furthermore, all participants were tested during the follicular phase of their menstrual cycle (days 3–10) to eliminate the potential confounding factor of differential emotional responding due to hormonal differences [28]. Although no participants wished to drop out of the study altogether, we only have partial data available for two of our participants in the BPD group; one did not wish to complete the BODY task and the other the PERSON task. Furthermore, we have the baseline disgust data points missing for 2 participants (one BPD and one MDD patient) in the BODY task, as they left these blank.

### Measures

#### Diagnostic interviews

The MINI is a short, structured, DSM-IV-based diagnostic interview, developed for use in research. The SCID-II is a semi-structured diagnostic interview to diagnose personality disorders according to DSM-IV Axis-II in research or clinical settings.

#### Symptom rating scales

The Personality Assessment Inventory-Borderline Features Scale (PAI-BOR; [29]) was used to measure BPD symptom severity. Twenty-four items, rated by the participant on a four-point scale (‘in general’: false, slightly true, mainly true, very true), assess four aspects of BPD symptomatology: affective instability, identity problems, negative relationships, and self-harm. The results can be interpreted using a cut-off score of 38, corresponding to a clinically significant threshold [30]. The PAI-BOR scale has been shown to specifically target BPD, such that in a study which included patients with other Axis I and Axis II disorders, BPD patients scored significantly higher than the clinical comparison sample [31]. We used the Hamilton Depression Rating Scale (HAMD; [32]) as an observer-rated measure of depressive symptom severity which has been shown to distinguish clearly between BPD and MDD patients [33].

#### The Self-relevant task (SRT)

The SRT is a semi-structured interview with two subtasks (PERSON and BODY tasks) that invite participants to reflect on the negative aspects of their person in general and their body in particular, respectively. Participants are invited to write a free narrative on the thoughts and emotions evoked by these subtasks in 3 minutes each time. Since previous literature suggested that body image may represent a necessary risk factor for NSSI in adolescents and that, in addition to emotion regulation, body-related pathology should be considered as a target when designing treatments for NSSI [34], we decided to investigate body-related emotions separately from those relating to personality and self-image on the whole.

The SRT was administered in a quasi-experimental design, with no control (non-self-relevant) condition and the PERSON task acting as the BODY task's control, in order to reduce task duration and total testing time. Both tasks were followed by the administration of visual analogue scales (VAS) to measure levels of disgust and change in level of self-harm urges. Before starting the tasks, the participants were given a printed description of the task and verbal explanation of the procedure. (**Diagram S1**. **Components of the SRT and their order.**) (**Text S1**. **Detailed description of task instructions**).

Since the BODY task was always performed subsequent to the PERSON task, it was essential to ensure that the participants returned to a baseline neutral emotional state. During the 5-minute washout period, the interviewer had an informal conversation with the participant, trying to take the participant's mind away from the task. To confirm that the patients returned to their baseline, VAS measurements of disgust levels were taken at the beginning of both tasks. The visual analogue scales were lines indicating a range of values, with the lowest value on the left (0 = “not disgusted at all”) and the greatest value on the right (10 = “extremely disgusted”). The participant marked the line to indicate how they felt.

After the experiment they were asked to rate on the same scales how thinking and writing about this subject made them feel. Changes in *self-harm urge (SHU) levels* were also measured after each writing session. Instead of measuring absolute levels, we asked participants to rate the change in their SHU levels relative to the level they had prior to the task on a −10 to 10 (“less” to “more”) visual analogue scale, where zero would indicate no change relative to baseline. For SHU, we decided to measure changes as opposed to pre- and post-task levels in order to avoid priming our participants to thoughts of SH.

The narratives produced by the participants during the 3-minute writing sessions were coded for labels of emotions. For each participant's script, the labels were counted and coded into the following categories: *basic emotions:* 1. anger, 2. anxiety, 3. disgust, 4. happiness, 5. sadness; *complex emotions (disgust-related):* shame/guilt; *other:* 1. worthlessness, 2. other non-specific negative emotions. Indirect labels were also recorded. For example, if the participants used longer, wordy descriptions to describe how they felt *(e.g. “made me feel not good at my job” [worthlessness])* or expressions which required some thought regarding where they belong, often making it necessary to examine the context of the expression too *(e.g. I feel frustrated [anger])*.

In addition to the five basic emotions, we decided to include further, complex emotions (shame, guilt, worthlessness), as they appeared common. We aimed to devise categories that would allow each expression only to belong to one category with the least amount of ambiguity. Although shame and guilt are distinguished from each other and have their own literature, respectively, we decided that for our purposes it was practical to merge them into one category, because they were often indistinguishable from the patients' scripts. Some labels found in the narrative still entailed some extent of ambiguity; the category membership of these was decided by consensus between three members of the research team without knowing the diagnostic group membership of the participant. The labels were counted and their numbers for each patient were entered into a table together with visual analogue scale rating changes in self-harm urges following the task. As all coding was carried out primarily by the main author, inter-rater reliability was not calculated.

### Procedure

Testing took place on a separate occasion from the diagnostic interview. (For a table illustrating the meetings schedule, see **Table 1**.) Observer-rated and self-report instruments were administered prior to the Self-relevant Task (SRT; see below), either on the same day as the diagnostic interview or on the day of testing. The two meetings took place either within the same follicular phase or were separated by 1–3 menstrual cycles. The test-retest reliability of the PAI-BOR observed in previous studies [35] [36] indicated that its results should be valid within a time window of this magnitude. Although the construct measured by the HAMD changes much faster than that measured by the PAI-BOR, we used the HAMD score mainly for the general description of the depressed group in terms of depression severity. In addition, where the two meetings took place in different menstrual cycles, the HAMD was repeated to reflect the current clinical picture. The two subtasks were always performed in the same order, starting with the PERSON task with a 5-minute washout period before the BODY task. Participants were given an honorarium of £6 per hour for their time and a contribution was made towards their travel expenses. Participants were debriefed after the testing session, and an appointment was offered to them with a consultant at the Complex Cases Service, if necessary, for the following day. The debriefing included a risk assessment, recapitulated the safety plan, and encouraged participants to report to us any unpleasant after-effects of the experiment. The general practitioner of each participant was informed about their patient's participation in the study. BPD patients received regular support on an outpatient basis from the Complex Cases as part of their normal care. One MDD patient requested an appointment with the consultant psychiatrist and one BPD patient requested support following the experiment, but no actual self-harm could be linked to the study.

**Table 1:** Meetings schedule.

Meeting 1	Meeting 2*
Diagnostic interviews: MINI & SCID	Questionnaires: HAMD**, PAI-BOR
Questionnaires: HAMD, PAI-BOR	Self-relevant Task
	Debriefing

*The time interval between the first and second meeting was 0–3 menstrual cycles.

**Repeated only if Meeting 2 not done in the same menstrual cycle.

### Ethics statement

The local NHS research ethics committee approved this research (Cambridgeshire 4 Research Ethics Committee, NHS National Research Ethics Service, reference number: 09/H0305/10). Written informed consent was obtained from each participant.

### Statistical analysis

The participant groups were compared using ANOVAs with regards to their demographic characteristics where variables followed a normal distribution. As most of our variables followed a non-normal distribution, we used non-parametric tests to carry out formal testing of statistical significance of group differences and associations of variables.

#### Hypothesis tests

The non-uniformity across diagnostic groups regarding baseline disgust (Kruskal-Wallis [KW] tests: PERSON task: whole sample: p<0.001; Mann-Whitney U (MWU) test: BPD vs. MDD: p = 0.176; BPD vs. HV: p = 0.001); BODY TASK: whole sample: KW: p = 0.011; MWU: BPD vs. MDD: p = 0.277; BPD vs. HV: p = 0.014) indicated a need to treat post-task disgust levels and change in disgust (post- minus pre- task disgust levels) on the VAS and disgust labels from the narrative as different types of variables: measures of overall disgust levels and disgust reactivity, respectively.

To investigate between-group differences regarding overall post-task disgust levels and disgust reactivity as measured by the VAS, we used the KW test and MWU test for pair-wise comparisons. In the case of disgust reactivity as measured by disgust labels from the narrative, associations between BPD status and disgust were tested first in a BPD+HV then in a BPD+MDD group, using Fisher's exact test on binarised values (for the rationale for and methods of binarisation, see below). To test for an association between overall post-task disgust levels and SHU change, Fisher's exact test between post-task VAS disgust levels and SHU change was carried out, using binarised values of the variables. Fisher's exact test was also used to check for an association between binarised values of disgust reactivity and SHU change, for both VAS disgust change and disgust labels from the narrative.

During the binarisation of SHU change values, the value of “1” was assigned if the patient indicated any value other than zero. Similarly, in case of emotion labels in the narrative, no mention at all of the given emotion in the task resulted in the code “0” and mentioning once or more was coded as “1”. As some increase in self-disgust on the VAS was reported by nearly all participants, self-disgust change was recoded as “1” only if the change was greater than the group median value. All changes less than the median value were recorded as “0”. The same procedure was carried out on post-task disgust levels using respective group median levels. The use of Fisher's exact test and such binarisation as opposed to using Spearman's test was felt appropriate; firstly, because scatter plots of the data suggested that if there is association between these variables, such association is unlikely to be of a simple linear nature; secondly, because values for emotion labels in the narrative were more accurately represented in a dichotomous fashion, because this variable was not continuous but took discrete values between 0 and 5 (with values of 2 and above being exceedingly rare). Furthermore, it seems very unlikely that there would be a simple linear relationship between emotion intensity and number of labels in the narrative, since some subjects might have reiterated points they made independently of the intensity of the emotion (e.g. they might feel they need to expand on their point, or want to fill out time available) whereas others may not.

To confirm that any significant findings were specific to disgust as opposed to generally to negative emotions, Fisher's exact test was performed in an explorative fashion on all the other negative emotions reported in the narrative.

#### Tests investigating task validity

To demonstrate the effectiveness and specificity of the SRT to generate SHU changes in BPD patients, the correlation between PAI-BOR scores and VAS measurements of SHU change were examined with Spearman's correlation test. To check for concurrence, Fisher's exact test was carried out between binarised measures of disgust as per the narrative and VAS disgust change. To examine the effectiveness of the washout procedure, baseline disgust levels in the PERSON and BODY tasks using the related samples Sign test was carried out.

## Results

### Demographic and clinical characteristics of the participant groups

Demographic and clinical characteristics of the three participant groups are shown in **Table 2**. The three groups were well-matched for age (ANOVA, F(2, 66) = 2.915; p = 0.061). The two patient groups both spent fewer years in education than the HV group, and they were not different from each other (ANOVA, F(2,66) = 9.065; p<0.001; post hoc comparison with the Bonferroni test: BPD vs. HV : p<0.001 and MDD vs. HV: p = 0.003). Thirteen of the 17 BPD patients and 16 of the 27 MDD patients, but none of the healthy participants, were taking some psychotropic medication. As expected, the three groups were different from each other on HAMD mean scores (ANOVA, F(2,51) = 92.307; p<0.001), with the BPD group having more depressive symptoms than the HV group (Bonferroni: p<0.001), but significantly less than the MDD group (Bonferroni: p<0.001) the mean total score value of which suggested moderately severe depression. The PAI-BOR scores reflected the fact that the BPD group had moderate to severe BPD and the MDD group also showed sub-threshold but significantly more symptomatology than the HV group did (ANOVA, F(2, 63) = 44.57; p<0.001; BPD>MDD>HV, each post hoc comparison with the Bonferroni test: p<0.001). The two patient groups were not different in terms of number of previous depressive episodes.

**Table 2:** Demographic and clinical characteristics of the diagnostic groups.

	BPD	MDD	HV	P value for ANOVA
**Number**	17	27	25	-
**Age**	35.35 (7.794)	35.41 (7.841)	30.68 (7.712)	0.061
**Education**	15.06 (2.657)	15.78 (3.093)	18.80 (3.476)	<0.001
**HAMD**	11.13 (6.428) n = 16	20.74 (4.126) n = 23	0.80 (0.941) n = 15	<0.001
**PAI-BOR**	46.65 (13.463)	28.41 (8.958)	15.27 (8.967) n = 22	<0.001
**Number of past depressive episodes**	4.40 (2.923) n = 15	4.52 (4.182)	N/A	-

Values are means and standard deviations. BPD: borderline personality disorder; MDD: major depressive disorder; HV: healthy volunteers; HAMD: Hamilton Depression Rating Scale; PAI-BOR: Personality Assessment Inventory - Borderline Features Scale.

### PERSON task

#### Differences in post-task disgust

Disgust levels post task were quite elevated, compared to baseline levels, in the two patient groups but not in the HV group (**Table 3**). Comparisons with the K-W test confirmed that there were significant between-group differences in post-task disgust levels (p<0.001); the BPD group reported higher levels than the HV group (MWU: BPD vs HV: p<0.001), but the patient groups were not significantly different from each other (MWU: BPD vs MDD: p = 0.414).

**Table 3:** Median values for VAS measures of disgust and self-harm urge level changes in the PERSON and BODY tasks.

	Measure	BPD (n = 16)	MDD (n = 27)	HV (n = 25)
**PERSON task**	**Baseline disgust level**	0.45	0	0
	**Post-task disgust level**	5.95	5.2	0.2
	**Disgust change**	2.45	4.1	0.2
	**SHU change**	1.3	0.0	0.0
**BODY TASK**	**Baseline disgust level**	0.4 (n = 15)	0.05 (n = 26)	0
	**Post-task disgust level**	7.95	5.3	0.8
	**Disgust change**	6.50 (n = 15)	4.50 (n = 26)	0.60
	**SHU change**	2.75	0	0

BPD: borderline personality disorder group; MDD: major depressive disorder; HV: healthy volunteer group; SHU: self-harm urge.

#### Differences in disgust reactivity

Median values for VAS self-disgust change were higher for both patient groups than for HVs (**Table 3** **.**). There were significant between-group differences (p = 0.001), with a significant difference between healthy controls and BPD patients (MWU: BPD vs HV: p = 0.009) but not the two patient groups (MWU: BPD vs MDD: p = 0.744). For a summary of emotions evoked by the PERSON task in our three subject groups as per the narrative analysis, please refer to **Table S1 (Emotions reported in the PERSON and BODY task narratives**). The narrative analysis revealed that the BPD group reported more disgust in their narratives than either control groups. Fisher's exact tests showed significant association between BPD status and reporting disgust both when looking at a subsample of BPD and healthy volunteers (p = 0.026) or one composed of the two patient groups (p = 0.005). It is of note that the reporting of no other negative emotion showed significant association with BPD status (BPD vs HV: anger p = 0.120; anxiety: p = 1.000; sadness: p = 0.215; worthlessness: p = 0.662; shame/guilt: p = 0.748; non-specific negative emotions: p = 1.000; BPD vs MDD: anger p = 0.111; anxiety: p = 0.494; sadness: p = 1.000; worthlessness: p = 0.494; shame/guilt: p = 0.752; non-specific negative emotions: p = 1.000).

#### Association between post-task disgust and SHU change

Fisher's exact tests between binarised SHU change and post-task VAS measures of disgust revealed no significant association (whole sample: p = 0.595; BPD: p = 1.000; MDD: p = 0.678; HV: p = 0.280).

#### Association between disgust reactivity and SHU change

Fisher's exact test indicated no significant association between binarised SHU changes and disgust reactivity as measured by change in disgust on the VAS, except in the MDD group (whole sample: 0.600; BPD: p = 0.569; MDD: p = 0.033; HV: p = 1.000). It is of note that the same test for association between binarised SHU changes and disgust mentioned in the narratives in the whole sample was highly significant (p = 0.009). At group level, though, the association was not significant (BPD: p = 0.245; MDD: not calculated due to lack of reported disgust; HV: p = 1.000). Interestingly however, in the BPD group, everyone who described disgust in their narratives (N = 5) also reported a change in their SHU levels. It is of note that seven out of 11 patients who did not describe disgust in their narratives also reported a SHU change. It seems that the significant association between narrative disgust and SHU level change at whole sample level was driven by the positive association in BPD, as reporting disgust was very rare in the other two groups (MDD: 0 out of 27; HV: 1 out of 25). Eight out of 27 MDD patients reported a SHU change; however, none described any disgust, consistent with a lack of correlation between disgust and SHU changes. Both disgust and SHU changes were very rare in the HV group, with one participant indicating each, respectively, and none both.

No other emotions were associated with SHU changes as shown by Fisher's exact tests on either whole sample or within group levels.

### BODY task

#### Differences in post-task disgust

**Table 3** shows the median values for the VAS measures in the BODY task. K-W tests revealed that there were significant between-group differences in post-task disgust levels (p<0.001). The BPD patients indicated more disgust than both the MDD and the HV group (MWU: BPD vs. MDD: p = 0.013; BPD vs. HV: p<0.001). Although this trend was present in the PERSON task, too, there the difference between the two patient groups was not significant, suggesting that perhaps the BODY task tapped into an area that is more sensitive for BPD than for MDD patients.

#### Differences in disgust reactivity

K-W tests were again performed to test for differences between the diagnostic groups as regards changes in self-disgust VAS measures (post- minus pre-task disgust). There were significant between-group differences (p<0.001), with the BPD group differing from the healthy controls (MWU: BPD vs. HV: p<0.001) but not from MDD patients (MWU: BPD vs MDD p = 0.108). The pattern of emotions evoked by the BODY task as per emotion labels in the narrative are shown in **Table S1 (Emotions reported in the PERSON and BODY task narratives)**. The BPD group reported more disgust than either control groups. Pairwise Fisher's exact tests revealed a significant association between BPD status and reporting disgust when looking at the BPD and healthy control group (BPD vs HV: p = 0.007) but not when testing for the same association in the two patient groups (BPD vs MDD: p = 0.526). No other negative emotion showed an association with BPD status (BPD vs HV: anger p = 0.084; anxiety: p = 1.000; sadness: p = 0.376; worthlessness: p = 0.332; shame/guilt: p = 1.000; non-specific negative emotions: p = 0.485; BPD vs MDD: anger p = 0.761; anxiety: p = 0.223; sadness: p = 0.229; worthlessness: p = 0.092; shame/guilt: p = 0.082; non-specific negative emotions: p = 1.000).

#### Association between post-task disgust and SHU change

A Fisher's exact test between binarised SHU change and post-task disgust VAS measures revealed no significant association (whole sample: p = 0.411; BPD: p = 0.569; MDD: p = 1.000; HV indicated no SHU change) in the BODY task.

#### Association between disgust reactivity and SHU change

A Fisher's exact test between change in disgust on the VAS and SHU level change showed no significant associations (whole sample: p = 0.781; BPD: p = 1.000; MDD: p = 0.645; HV: no change in SHU).

In terms of the narrative measures, it is worth noting that more BPD patients described disgust in the context of the BODY than the PERSON task (1/2 vs. 1/3). Identical to the PERSON task, Fisher's exact test between SHU changes and disgust labels from the narrative was significant (p = 0.002) in the whole sample level analysis, but not when looking at the groups on their own (Fisher's exact test: BPD: p = 0.569; MDD: p = 0.153; HV: no SHU reported). Despite the lack of a statistically significant result, upon investigating the data it was apparent that similarly to the PERSON task, in the BPD group the overwhelming majority of those who described disgust (7 out of 8) also reported SHU level changes. However, those who did not describe disgust also often reported SHU level changes (5 out of 8). Unlike in the PERSON task, in the BODY task, a significant proportion of MDD patients also described disgust (10 out of 27), and these patients were several times more likely to report SHU level changes too (4 out of 10 vs. 2 out of 17). Healthy volunteers, again, reported disgust very rarely (2 out of 25) and none of them reported any SHU level change. Overall, the significant association between narrative disgust and SHU level changes in the BODY task was likely driven by the BPD and MDD groups, even though the association failed to reach statistical significance in the BPD group.

All other negative emotions reported in the narratives were non-significant with regards to their association with SHU change.

### Tests of task validity

#### PERSON task

There was a strong correlation between PAIBOR score and SHU change (rho = 0.405, p = 0.001) at whole sample level, and in the BPD group (rho = 0.507 p = 0.045), but not in the other two groups. When looking at the two patient groups combined, the association remained significant (rho = 0.341; p = 0.025). Contrary to expectation, Fisher's exact test between the binarised VAS measure of disgust change and that of narrative disgust did not reveal a significant association neither at whole sample level (Fisher's p = 0.673) or at group level (BPD: p = 1.000; HV: p = 1.000; none of the MDD participants mentioned disgust).

#### BODY task

P values for Spearman's correlation tests indicated a highly significant correlation between PAI-BOR score and SHU change on the whole sample level (ρ = 0.504; p<0.001), but the association failed to reach significance when looking within the BPD group (rho = 0.375 p = 0.153). However, when looking at the two patient groups together (excluding the healthy volunteers), a significant positive correlation was still apparent (ρ = 0.439; p = 0.003). Like in the PERSON task, Fisher's exact test between binarised VAS measure of disgust change and disgust as measured by narrative labels did not reveal a significant association at either whole sample (Fisher's p = 0.101) or group level (BPD: p = 1.000; MDD : p = 0.226; HV: p = 0.150).

#### Washout

To test the effectiveness of the washout procedure, we compared baseline disgust levels measured before the PERSON task and the BODY task respectively, using the Sign test for related samples. The non-significant p-values (whole sample: p = 0.860; BPD: p = 0.549, MDD: p = 1.000; HV: p = 0.688) indicated that disgust levels were not higher when starting the BODY task than before the PERSON task, suggesting that any disgust generated in the PERSON task did not get carried over into the BODY task.

## Discussion

Based on previous work describing a variety of negative emotions prior to self-harming, we attempted to model the generation of self-harm urges, by asking our participants in a safe and controlled environment to focus on negative aspects of their person and body. Having a model would make it possible to identify risk factors and to test potential treatments. Our findings are in agreement with previous studies which identified trait measures of negative body attitudes and body shame (which, importantly, is also a disgust-related complex emotion) to be correlated with self-harming [37] [23]. Others [38] found elevated disgust proneness compared to healthy controls specific to certain domains in patients with BPD, with the biggest differences observed for self-disgust. We hoped that having a task based on focussing on one's own self would mimic real life processes and allow for high ecological validity in our design.

The SRT was successful at inducing disgust in both patients and healthy volunteers and in inducing self-harm urges in our patient groups (**Table 3**). Although the association between SHU changes and PAI-BOR scores seen in both tasks on a whole sample level was only seen within the BPD group in the PERSON (but not the BODY) task, the association remained significant when looking at the two patient groups merged. Overall, it seems that the task was not only successful at eliciting changes in self-harm urge levels but also that this response was linked to borderline symptom severity. The washout period seemed to work well and there was no evidence for any carry-over effects. Contrary to expectation, there was no association between the two measures of disgust reactivity we used: disgust labels from the narrative and disgust change on the VAS. As it has been pointed out in the context of BPD research [39], “when different methods produce discrepant results, several interpretations are possible”. The first logical interpretation is measurement error (i.e., the possibility that one or more methods are unreliable), but it is also possible that the methods differ in the precise construct they measure. Apart from measurement issues, other possibilities also exist. For example, distinct emotional response systems (e.g. experiential/cognitive, and motoric/behavioural) may respond differentially to the same stimuli, and such differences in response might not be caused entirely by measurement error.

As regards our hypotheses, when looking at the pattern of overall post-task disgust, it is apparent from the post-task VAS measures that while all three diagnostic groups exhibited higher disgust levels post task, the patient groups responded more strongly (**Table 3**). In the PERSON TASK **our first hypothesis** regarding the BPD patients reporting more self-relevant disgust in the context of focusing on the negative aspects of themselves was only partially confirmed; they were different from healthy volunteers but not from depressed patients. Recent literature [40] suggests that elevated self-disgust is not a disorder-general phenomenon, as patients with eating disorders and BPD score higher for both personal and behavioural disgust than those with other mental disorders. Our findings were consistent with this idea in the BODY TASK which distinguished the BPD patients from both comparison groups.

When looking at disgust reactivity, we saw a similar picture with our VAS measure; providing only partial confirmation for our **second hypothesis**, BPD patients were no different from the depressed on either task. This was contrary to our expectations, as we expected the BPD group to respond more strongly relative to the MDD group. The presence of a statistically significant difference between the BPD and both control groups in the BODY task with regard to post-task disgust levels supports the idea that despite the inability of the VAS measurement to pick it up when looking at changes from baseline, a difference between MDD and BPD patients may exist.

It must be also noted however, that the pattern of the disgust labels, which we included as an alternative measure for disgust reactivity, revealed that the frequency of disgust reported in the BPD group was also above that in the MDD group, and especially so in the PERSON task. Formal statistics in the PERSON task showed a significant difference when comparing BPD patients to healthy controls, but the significance was only marginal when comparing them to MDD patients, even though there were actually no MDD patients who reported disgust in this task at all. Our hypothesis however was only partially confirmed in the BODY task, where no significant difference between the two patient groups could be observed. Taken together, our results suggest that BPD patients have more baseline self-relevant disgust and respond with more disgust to focussing on negative aspects of themselves than healthy volunteers do but not necessarily more than depressed patients do. Our results allow for the proposition that differences in baseline levels as well as the magnitude of the response to the task may contribute to such differing outcomes across diagnostic groups.

Whilst our **third hypothesis** about an association between post-task levels of self-relevant disgust and changes in self-harm urge levels was not confirmed in either task, our **fourth hypothesis** about a similar association between measures of disgust reactivity (as opposed to absolute levels of self-relevant disgust) and SHU level changes was partially confirmed. In the PERSON task, there was an association between post-task self-disgust level and SHU changes in the MDD group, but this was not present at whole sample level or in the BPD group, nor was it replicated in the BODY task in any group. It is possible that this result was due to the greater number of participants in the MDD than in the BPD group where the small sample size may not have allowed for sufficient power for the tests, although a whole sample level analysis did not show a significant association either. An alternative explanation could be that our VAS scale measurements were not sensitive and accurate enough to detect such an association, which would be consistent with the lack of correlation between the narrative and VAS measures. This latter explanation is supported by the fact that when considering data on disgust extracted from the narratives, it turned out that disgust labels were indeed significantly associated with SHU level changes in both tasks on a whole sample level. Although within-group analyses revealed no statistically significant association, closer investigation of the data hinted at the association being driven by the positive association in the BPD group in the PERSON task and by both patient groups in the BODY task. As with our previous hypotheses, it appears that, in our experiment, the participants' narrative was a more sensitive measure. It is of importance that disgust was the only emotion associated with SHU changes, indicating that the associations are not likely to be explained by a general link between SHU changes and negative emotions. Although self-disgust has been linked to self-harm in a number of studies [38], [40] and in a review on self-harm for practitioners [19], the literature on the exact nature of this relationship is currently rather limited. In a retrospective study of 101 female patients with BPD investigating motives for non-suicidal self-injury [20], participants indicated disgust as one of the emotions characterising their feeling state before self-harming with a frequency between “sometimes” and “frequently”. Our findings provide further evidence from a quasi-experimental manipulation that hints at the possibility of a link between self-relevant disgust and self-harm urges. Further research is needed however to clarify the nature of this possible link.

In our experiment, the BODY subtask seemed to elicit a somewhat different pattern of responses in terms of the elicited emotions as per the narratives. The correlation between body-related narrative disgust and self-harm urge changes is not inconsistent with previous findings from studies in adolescents. Body image has been identified as a moderator of non-suicidal self-injury [41], it has been found to be a sufficient but not necessary factor which indirectly influences SH behaviour through depressive symptoms [23], and those who had made a suicide attempt reported more negative body attitudes and experiences than non-suicidal patients or healthy controls did [42].

### Limitations and future directions

One of the limitations of our study related to the relatively small sample sizes, commensurate with a pilot study, especially in the BPD group. This might have meant that some of the statistical tests may have failed to find a statistically significant difference or association due to lack of power, although some general patterns were clear from the descriptive statistics. The unfortunate fact that the smallest sample size was in our group of main interest is at least partially counter-balanced by one of the major strengths of study, namely the diagnostic precision and diagnostic “purity” of our patient samples. Of course, our very strict inclusion criteria meant a compromise, making it more difficult to obtain a larger sample.

Another important limitation of our study was the lack of a suitable control condition. Although an ideal control condition would have been a task with a similar layout and duration, where instead of writing about oneself, the participants could have been invited to write about someone else they know. Such a control condition would have allowed us to check for the specificity of self-targeted emotions in the changes observed during and after the self-relevant tasks. However, due to time constraints, further lengthening the procedure was not an option. Nevertheless, in this semi-experimental design, one may regard the two tasks as each other's controls. Although it is easily apparent that participants did not respond identically to the two tasks, most of our main hypotheses were confirmed or otherwise by both tasks. Our intention in this initial pilot was not to establish the specificity of any changes in mood to the new task but to carry out some feasibility evaluations, including establishing the task's effectiveness in inducing negative emotions, including self-disgust, and SHU, as well as its acceptability, before using it in a larger sample. The SRT showed reliable performance in BPD, without causing any untoward incident.

Although the debriefing session following the SRT ensured the safety of participants, the lack of a second washout and subsequent VAS measurements is a limitation of the study with regards to establishing the duration of the effects of the task. Although the participants did not have clinically significant residual self-harm urges, we cannot say for certain that their self-harm urge levels returned to baseline by the end of the debriefing. Further work is needed to establish the duration of the effects of the SRT.

The fact that our patients spent fewer years in education than our healthy volunteers did was not at all surprising, considering the impact of BPD and recurrent depressive episodes (as shown in **Table 2**) on social-occupational functioning, including academic performance. A further limitation of our study is that we only included female participants; therefore, our findings may only apply to females.

A relatively minor weakness in our analysis of the narrative responses was merging together the categories of shame and guilt. These two emotions have been conceptualized as being disgust-related complex emotions but they also have some important differences. Therefore, at first we tried to record them separately, however, we found that they were often very difficult to distinguish in the patient narratives. This is probably not so surprising considering their shared characteristics. Similarly, we did not distinguish between suicidal and non-suicidal self-injury. However, it is likely that most of the SHU changes elicited were non-suicidal.

It would be worthwhile to replicate these preliminary findings with a task design advised by our current findings. More VAS scales could be provided to capture not only disgust but other self-relevant emotions too, in order to avoid a potential tendency for participants to use the self-disgust scale to report various negative emotions they feel as disgust. More detailed descriptions as anchor points on the VAS scales may improve their reliability.

Although using a SHU change measure as opposed to measuring absolute levels before and after writing the narrative might have imposed a negative recall bias in our design, it was necessary in order to avoid priming our participants to thoughts of SH by asking them to rate their SH urge levels in advance. Although the fact that we measured self-reported SHU level changes as opposed to actual SH behaviour could be considered a limitation, we know that actual self-harm often follows thinking about self-harm. We also know that BPD patients also often present to the health service with self-harm thoughts. Currently, we have no specific acute treatment to offer for these. Self-harm thoughts are highly unpleasant and identifying factors that are linked to them and could be targeted by new treatments would be an important therapeutic breakthrough. Eliciting actual self-harm behaviour would be unethical. Although the question as to whether findings with induced self-harm urges can be extrapolated to actual self-harm behaviour would need to be confirmed, it seems that the BODY subtask of the SRT has the potential to be developed into a standardised psychological challenge for use in experimental trials of pharmacological or psychological interventions to treat self-harm behaviour. Testing dose-response relationships and whether it is possible to bring participants to the same tolerable subjective level would be the next step in the development of a challenge for use in treatment trials. Both patient groups responded well to the task, and, although a significant proportion of them developed self-harm urges, no patient went on to harm herself.

## Conclusions

This paper reports on initial findings in patients with borderline personality disorder, major depressive disorder, and healthy volunteers using narrative and visual analogue scale measures during a newly designed task aiming to elicit changes in one's emotional state through focusing on one's own self. The task was designed to function as an emotional challenge and was expected to lead to an increase in self-relevant negative emotions and self-harm urge levels (but no actual self-harming behaviour). With this challenge, we attempted to model the generation of self-harm urges. Having a model would make it possible to identify risk factors and to test potential treatments.

The task performed well in terms of eliciting an increase in self-relevant negative emotions, including disgust, and self-harm urge levels in BPD and MDD, with less of an emotional change and no increase in self-harm urge levels in healthy volunteers. Further work is necessary to establish the specificity of these findings and other task characteristics.

Reporting self-disgust (but not any of the other emotions studied) in the narrative produced during the task was significantly associated with an increase in self-harm urge levels at whole sample level. Although similar associations could not be confirmed with visual analogue scale measures of self-disgust, it is important to note that these two measures of self-disgust may have tapped into related but not identical constructs.

Taken together, our results suggest that BPD patients have more baseline self-relevant disgust and respond with more disgust to focussing on negative aspects of themselves than healthy volunteers, and that these heightened disgust reactions and states might be associated with increased self-harm urges. However, it is possible that these findings are not unique to BPD but also apply to similar conditions, such as major depressive disorder.

## Supporting Information

Table S1 **Emotions reported in the PERSON and BODY task narratives.**
(DOCX)

Diagram S1 **Components of the SRT and their order.**
(PDF)

Text S1 **Detailed description of task instructions.**
(DOCX)
