# Supplementary material for: Self-Relevant Disgust and Self-Harm Urges in Patients with Borderline Personality Disorder and Depression: A Pilot Study with a Newly Designed Psychological Challenge
Source: PLoS One. 2014 Jun 23;9(6):e99696. doi: 10.1371/journal.pone.0099696 (PMC4067282; doi:10.1371/journal.pone.0099696)
Supplement: Table S1 — Emotions reported in the PERSON and BODY task narratives. (DOCX) [file pone.0099696.s001.docx]

**Table S1. Emotions reported in the PERSON and BODY task narratives**

| **PERSON TASK** | **% of participants who reported the emotion** | **BPD (n=16)** | **MDD (n=27)** | **HV (n=25)** | **Total (n=68)** |
| --- | --- | --- | --- | --- | --- |
|  | Anger | 62.5 | 33.3 | 36.0 | 41.2 |
|  | Anxiety | 18.8 | 29.6 | 16.0 | 22.1 |
|  | Sadness | 6.3 | 7.4 | 24.0 | 13.2 |
|  | Disgust | 31.3 | 0 | 4.0 | 8.8 |
|  | Happiness | 0 | 0 | 0 | 0 |
|  | Worthlessness | 18.8 | 29.6 | 12.0 | 20.6 |
|  | Shame /Guilt | 50 | 40.7 | 40.0 | 42.6 |
|  | Non-specific negative | 50 | 48.1 | 52.0 | 50.0 |
|  | **Average** | **29.7** | **23.6** | **23.0** | **24.8** |
| **BODY TASK** | Anger | 50.0 | 55.6 | 20.0 | 41.2 |
|  | Anxiety | 6.3 | 25.9 | 12.0 | 16.2 |
|  | Sadness | 6.3 | 22.2 | 20.0 | 17.6 |
|  | Disgust | 50.0 | 37.0 | 8.0 | 29.4 |
|  | Happiness | 0 | 0 | 0 | 0 |
|  | Worthlessness | 43.8 | 18.5 | 28.0 | 27.9 |
|  | Shame / Guilt | 31.3 | 7.4 | 28.0 | 20.6 |
|  | Non-specific negative | 37.5 | 40.7 | 24.0 | 33.8 |
|  | **Average** | **28.2** | **25.91** | **17.5** | **23.3** |

Values are percentages of participants who used the emotion label in question at least once in their narrative. Average refers to the average percentage of reporting an emotion.

BPD: borderline personality disorder; MDD: major depressive disorder; HV: healthy volunteers
