# Supplementary figures and images for: Self-Relevant Disgust and Self-Harm Urges in Patients with Borderline Personality Disorder and Depression: A Pilot Study with a Newly Designed Psychological Challenge
Source: PLoS One. 2014 Jun 23;9(6):e99696. doi: 10.1371/journal.pone.0099696 (PMC4067282; doi:10.1371/journal.pone.0099696)

# Components of the self-relevant task

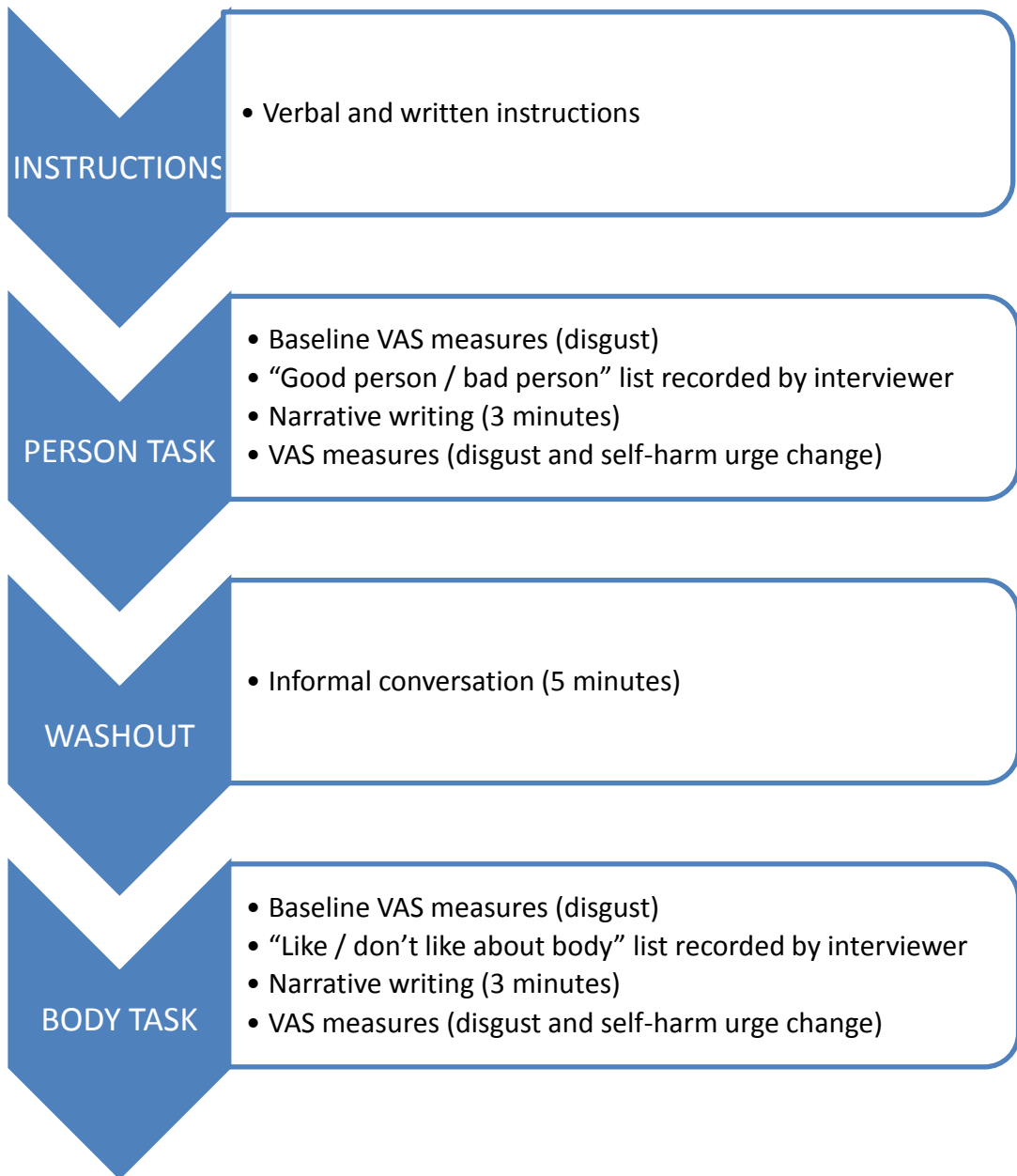

Supplement: Diagram S1 — Components of the SRT and their order. (PDF) [file pone.0099696.s002.pdf]
