# Supplementary material for: Self-Relevant Disgust and Self-Harm Urges in Patients with Borderline Personality Disorder and Depression: A Pilot Study with a Newly Designed Psychological Challenge
Source: PLoS One. 2014 Jun 23;9(6):e99696. doi: 10.1371/journal.pone.0099696 (PMC4067282; doi:10.1371/journal.pone.0099696)
Supplement: Text S1 — Detailed description of task instructions. (DOCX) [file pone.0099696.s003.docx]

**Text S1. Extract of the instructions sheet for the SRT tasks**

Subtask 1: Thinking of oneself as a person (PERSON task)

‘*Overall, do you think of yourself as a good person or a bad person? OK, tell me some things about yourself that make you think of yourself as a good person* (interviewer records the list). *OK, now tell me some things about yourself that make you think of yourself as a bad person* (interviewer records the list).

*When you think about things you said that make you feel you are a bad person, which one makes you feel the worst? Can you describe how that makes you feel?’*

The participant was then allowed three minutes to write about the last question producing the narrative that was later analysed.

Each participant received the following instructions when writing the script:

*“Can you remember the worst example of this happening/causing you suffering? Give us as many details as you can. How does it make you feel? What sensations can you feel in your body when you think of this thing?”*

Subtask 2: Imagining one’s own body (BODY task)

*‘Now think of your own body as a whole. How would you describe it? OK, tell me some things that you like about your body (*interviewer writes down list*). Now, tell me some things that you don’t like about your body (*interviewer writes down list*). When you think about the things you said you don’t like about your body, which one makes you feel the worst? Can you describe how that makes you feel?’*

The participant was then allowed three minutes to write about the last question producing the narrative that was later analysed.

Each participant received the following instructions when writing the script:

*“What does it look/sound/smell/feel like? Give us as many details about this bad thing as you can. How does it make you feel? What sensations can you feel in your body when you think of this thing about your body?”*
